# Effect of presurgical aerobic exercise on cardiometabolic health 30 days after bariatric surgery

**DOI:** 10.14814/phy2.15039

**Published:** 2021-10-29

**Authors:** Nicole M. Gilbertson, Natalie Z. M. Eichner, Julian M. Gaitán, Mahnoor Khurshid, Elizabeth A. Rexrode, Sibylle Kranz, Peter T. Hallowell, Steven K. Malin

**Affiliations:** ^1^ Department of Kinesiology Pennsylvania State University Altoona Pennsylvania USA; ^2^ Department of Kinesiology University of Virginia Charlottesville Virginia USA; ^3^ Department of Surgery University of Virginia Charlottesville Virginia USA; ^4^ Department of Kinesiology and Health Rutgers University New Brunswick New Jersey USA; ^5^ Division of Endocrinology, Metabolism and Nutrition Rutgers University New Brunswick New Jersey USA; ^6^ New Jersey Institute for Food, Nutrition and Health Rutgers University New Brunswick New Jersey USA; ^7^ Institute of Translational Medicine and Science Rutgers University New Brunswick New Jersey USA

**Keywords:** arterial stiffness, body composition, fitness, inflammation, quality of life

## Abstract

We evaluated the effect of preoperative standard medical care (SC) vs. unsupervised aerobic exercise combined with SC (EX + SC) on cardiometabolic health and quality of life (QoL) 30 days after bariatric surgery. Bariatric patients (*n* = 14, age: 42.3 ± 2.5 years, body mass index: 45.1 ± 2.5 kg/m^2^) were match‐paired to presurgical SC (*n* = 7) or EX + SC (*n* = 7; walking 30 min/day, 5 day/week, 65–85% HR_peak_) for 30 days. Body composition, peak cardiorespiratory fitness (VO_2_peak), QoL, inflammation (adiponectin, leptin, cytokeratin‐18), and a 120 min mixed meal tolerance test was performed to assess aortic waveforms (augmentation index, AIx@75), insulin sensitivity, and glucose total area under the curve (tAUC) at the time of surgery (post‐intervention) and 30 days post‐surgery. EX + SC had significantly higher high molecular weight (HMW) adiponectin (*p *= 0.01) and ratio of HMW to total adiponectin (*p *= 0.04) than SC at 30 days post‐surgery, although they significantly (*p *= 0.006; ES = 1.86) decreased total time spent in moderate to vigorous physical activity (MVPA). SC had a significantly greater increase in VO_2_peak (*p *= 0.02; ES = 1.54) and decrease in 120 min AIx@75 (*p *= 0.02; ES = 1.78) than EX + SC during the post‐surgical period. The increase in MVPA was associated with a reduction in cytokeratin‐18 (*r* = −0.67, *p *= 0.02). Increased VO_2_peak was associated with increased activity/mobility QoL domain (*r* = 0.52, *p *= 0.05) and decreased 120 min AIx@75 (*r* = −0.61, *p *= 0.03) from surgery to post‐surgery. Preoperative EX + SC did not maintain more favorable cardiometabolic health 30 days post‐operation in this pilot study. However, changes in MVPA appear important for QoL and should be considered in future work.

## Graphical abstract

Patients completing presurgical aerobic exercise did not sustain this behavior after surgery, and preoperative exercise did not cause more favorable improvements in cardiometabolic health 30d postoperation compared to patients receiving standard medical care. This research showed that increased fitness was important for quality of life in patients after bariatric surgery. Further, increased physical activity after surgery was linked to reduced inflammation in patients.

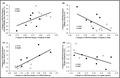

## 1 INTRODUCTION

Bariatric surgery is an effective cardiometabolic treatment for individuals with obesity. In fact, bariatric surgery induces significant decreases in body weight (Karamanakos et al., 2008; Pories et al., 1987; Rizzello et al., 2010; Streese et al., 2019), fat mass (Maïmoun et al., 2019), waist circumference (Streese et al., 2019), fasting glucose (Rizzello et al., 2010), blood lipids (Nicolás, 2015), inflammation (Swarbrick et al., 2006, 2008), blood pressure (Hinojosa et al., 2009; Streese et al., 2019), and arterial stiffness (Oliveras et al., 2017) as well as significant increases in insulin sensitivity (Rizzello et al., 2010; Samat et al., 2013) and quality of life (QoL) (Livingston and Fink, 2003; Peluso and Vanek, 2007) approximately 30 days after surgery. Unfortunately, not all patients undergoing bariatric surgery achieve these improvements in cardiometabolic health (Gilbertson et al., 2017).

A low calorie diet is current standard medical care (SC) prior to bariatric surgery to reduce liver size prior to surgery and improve surgical outcomes (Colles et al., 2006; Edholm et al., 2011). Aerobic exercise has proven to be an effective intervention to improve cardiometabolic health in obese adults as well as other clinical populations (2018 Physical Activity Guidelines Advisory Committee, 2018; Lin et al., 2015), and recently aerobic exercise has gained attention for not only reducing surgical risk, but also increasing cardiometabolic health and QoL in bariatric patients (Baillot et al., 2013, 2016; Gilbertson et al., 2020a, 2020b). We recently showed that individuals completing 30 days of aerobic exercise combined with standard care (EX + SC) prior to bariatric surgery had a shorter length of hospital stay than individuals undergoing SC (Gilbertson et al., 2020b). Interestingly, the rise in aerobic fitness after this EX + SC intervention was also associated with a shorter operating time and length of stay following surgery as well as improvements in lean mass, the ratio of circulating high molecular weight (HMW) to total adiponectin, and omental fat leptin gene expression (Gilbertson et al., 2020b). Limited work has evaluated the effect of pre‐bariatric surgery exercise on post‐surgical outcomes in patients, although Baillot et al. (2018) reported that bariatric patients who undergo presurgical exercise training improved physical activity and submaximal physical fitness to a greater extent than bariatric patients undergoing SC 1 year after bariatric surgery (Baillot et al., 2018). However, to date, it is unknown if adding aerobic exercise to preoperative SC enhances cardiometabolic health and QoL 30 days after bariatric surgery above and beyond SC. Moreover, it is unknown if preoperative exercise habits would persist in the post‐exercise period. Therefore, the purpose of this exploratory study was to evaluate the effect of preoperative EX + SC vs. SC alone on cardiometabolic health and QoL 30 days after bariatric surgery. We tested the hypothesis that EX + SC would enhance cardiometabolic health and QoL 30 days after bariatric surgery compared to SC alone.

## 2 MATERIAL AND METHODS

### 2.1 Study participants

**FIGURE 1:**
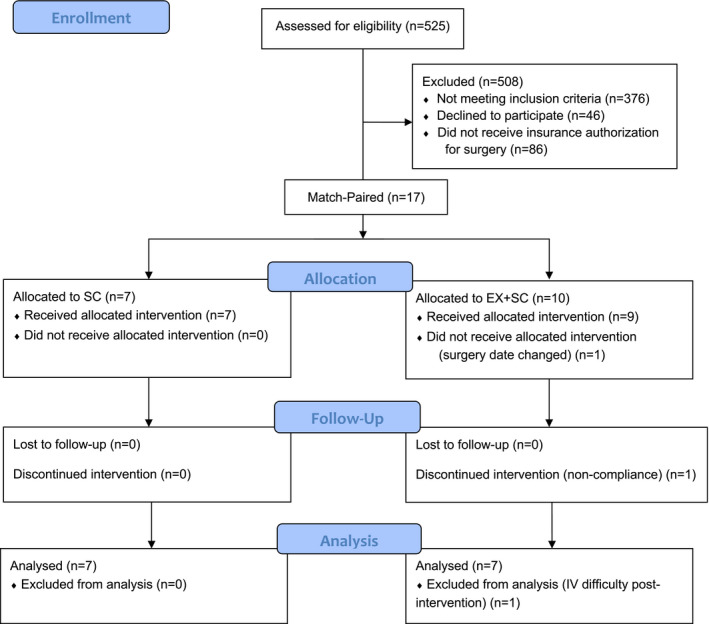
Consort flow diagram

This is an exploratory study and previously published methods are briefly outlined here for ease of the reader (Gilbertson et al., 2020a, 2020b). In short, participants were included in the study if they were 18–70 years old undergoing their first Roux‐en‐Y gastric bypass (RYGB) or sleeve gastrectomy (SG) procedure, not pregnant or lactating, and not taking medications known to alter body weight. Participants were excluded if they were physically active (>60 min/week of exercise), diagnosed with insulin‐dependent diabetes, had a history of cardiovascular disease, or diagnosed with cancer (<5 years) (Figure 1). Physician clearance for participation in the study and all surgical procedures were completed by one investigator (P.T.H.). Participants were match‐paired between EX + SC or SC based on body mass index (BMI), sex, race, and surgery type (RYGB or SG). Outcomes were assessed pre‐intervention, ~2 days before surgery (i.e., post‐intervention), and 30 days post‐surgery (Gilbertson et al., 2020a, 2020b). Thirty days post‐bariatric surgery is commonly studied in patients to evaluate the short‐term effect of bariatric surgery on cardiometabolic health (Karamanakos et al., 2008; Livingston and Fink, 2003; Rizzello et al., 2010; Samat et al., 2013; Streese et al., 2019). In addition, 30 days follow‐up is routine care at many bariatric surgery facilities, thereby increasing generalization of our findings to the medical community. Participants provided written and verbal informed consent as approved by the University of Virginia Institutional Review Board. The work described herein has been carried out in accordance with the Helsinki Declaration of 1975 as revised in 2008 and registered (Clinical Trial Registration Number: NCT03854981).

### 2.2 Body composition and cardiorespiratory fitness

Body weight and height were measured on a digital scale and stadiometer, respectively, to assess BMI. Air displacement plethysmography (BodPod) was used to determine body fat and fat free mass (FFM). Waist circumference was measured 2 cm above the umbilicus using a flexible tape measure. VO_2_peak was tested using indirect calorimetry on a treadmill (Carefusion, Vmax CART). Participants self‐selected a speed and grade was increased 2.5% every 2 min until volitional exhaustion as reported before (Gilbertson et al., 2020a, 2020b).

### 2.3 Non‐exercise and total physical activity

Accelerometers (Actigraph GT3X+, Pensacola, FL) were used to assess non‐exercise physical activity for 1 week prior to the mixed meal tolerance test (MMTT) at each time point. The Freedson VM3 (2011) algorithm was used to determine the percent of wear time spent in sedentary, light physical activity, or in moderate to vigorous physical activity (MVPA) per day (Sasaki et al., 2011). Individuals in EX + SC were instructed to remove their accelerometer during exercise. Total physical activity was determined by adding exercise time recorded by the A300 Polar fitness and activity trackers to accelerometer wear time at the post‐intervention time point.

### 2.4 Usual dietary intake and analysis

Ad libitum food intake was assessed using 3‐day food logs, including two weekdays and one weekend day. Participants were provided with reference guides and also given detailed instructions for recording food and beverages consumed. Intake reports were analyzed for 3‐day average energy, food groups, and nutrient consumption using Food Processor Nutrition Analysis Software (ESHA Research, Version 11.1).

### 2.5 Mixed meal tolerance test

Participants were educated to avoid alcohol, caffeine, dietary supplements, medication, and exercise for 24 h prior to testing (Gilbertson et al., 2020a, 2020b). Following an overnight fast, participants were admitted at approximately 8:00 a.m. to the Clinical Research Unit. Participants laid supine undisturbed for about 5 min to determine the average of three resting heart rate and blood pressure (BP) recordings using Dinamap (CARESCAPE V100 monitor, GE Healthcare). Fasting blood was collected to measure glucose, insulin, triglycerides (TG), high‐density lipoproteins (HDL), low‐density lipoproteins (LDL), total cholesterol, high sensitivity C‐reactive protein (hs‐CRP), cytokeratin‐18 (CK18), leptin, high molecular weight (HMW) adiponectin, and total adiponectin. A MMTT was then administered in which participants consumed 4 fl. oz. of an Ensure Plus shake (CHO 25 g, fat 5.5 g, protein 6.5 g). Circulating glucose and insulin were collected every 30 min up to 120 min after consumption of the mixed meal to assess glucose/insulin total area under the curve (tAUC) and insulin sensitivity (Matsuda and DeFronzo, 1999). Biochemical analyses were previously outlined (Gilbertson et al., 2020a, 2020b). Indirect calorimetry (Carefusion, Vmax CART) with a ventilated hood was used to determine resting metabolic rate. Respiratory exchange ratio (RER) was measured at 0, 60, and 120 min of the MMTT to estimate metabolic flexibility (i.e., the average of postprandial RER minus fasting RER). Fasting and postprandial augmentation index corrected to a heart rate of 75 bpm (AIx@75) was also measured by aplanation tonometry to reflect aortic waveforms (SphygmoCor® system, AtCor Medical) at 0, 60, and 120 min of the MMTT while patients rested quietly in the supine position. AIx@75 tAUC was determined using the trapezoid method.

### 2.6 Quality of life

The Laval questionnaire was used to assess QoL including: symptoms, sexual life, activity/mobility, personal hygiene/clothing, emotions, and social interactions domains as previously reported (Gilbertson et al., 2020a, 2020b). Scores for each domain were added and total scores analyzed; a higher score reflects better QoL.

### 2.7 Interventions

SC required patients attended a presurgical visit approximately 40 days prior to bariatric surgery. Patients were instructed by registered dieticians to consume a meal replacement shake for breakfast and lunch, snacks of raw vegetables, a dinner composed of 4 oz. of lean protein and steamed vegetables, and sugar‐free beverages for 2 weeks prior to surgery. Participants in EX + SC also completed at home walking for 30 min/day, 5 days/week, 65–85% heart rate peak (HR_peak_)for 30 days. The highest heart rate value achieved during the VO_2_peak test was recorded as HR_peak_ and used for the exercise prescription of EX + SC. Participants were required to complete 80% of exercise sessions, and A300 Polar fitness and activity trackers (Kempele, Finland) were used by participants and monitored by the research team to ensure adherence to the exercise prescription. The research team also conducted weekly check‐ins during the intervention by texting, emails, and/or phone calls with study participants in SC and EX + SC. Participants were provided with no instructions or recommendations for exercise in the 30 days after surgery.

### 2.8 Statistical analysis

It was determined that five obese adults would be needed to show the effect of short‐term EX on AIx@75 (delta of 782, SD of 397 with 80% power and alpha of 0.05) (Eichner et al., 2019) and insulin sensitivity (delta of 0.5, SD of 0.9 with 80% power and alpha of 0.05) (Kelly et al., 2012). Data were analyzed using SPSS Version 26 (IBM Analytics). Baseline (i.e., pre‐intervention) and time of surgery (i.e., post‐intervention) data has previously been reported (Gilbertson et al., 2020a, 2020b) but reported herein for ease. Normality was assessed using Shapiro–Wilk tests. Outliers were set as >2 standard deviations from the mean for all variables, and outliers were excluded for fasted AIx@75 (EX + SC *n* = 2), total adiponectin (SC *n* = 1), HMW adiponectin (SC *n* = 1), HMW:total (EX + SC *n* = 1), fasting glucose (EX + SC *n* = 1), 120 min glucose (EX + SC *n* = 1), HDL cholesterol (EX + SC *n* = 1), LDL cholesterol (EX + SC *n* = 1), total cholesterol (EX + SC *n* = 1), 0 min RER (EX + SC *n* = 1), insulin sensitivity (SC *n* = 2), fiber (SC *n* = 2), sugar (SC *n* = 1, EX + SC *n* = 1), trans fat (EX + SC *n* = 1), and physical activity variables (SC *n* = 1, EX + SC *n* = 1). Independent samples *t*‐tests evaluated differences between groups in the change from surgery to post‐surgery as well as at 30 days post‐surgery values for all variables except QoL and non‐normally distributed variables in which a non‐parametric Mann–Whitney *U* Test was used. Cohen's d effect sizes (ES) were also calculated on the interaction of treatments. ES relevance was interpreted as small (*d* = 0.2), medium (*d* = 0.5), or large (*d* = 0.8). Pearson's correlation was used to assess associations. Significance was set at *p* ≤ 0.05. Data are presented as mean ± SEM.

## 3 RESULTS

### 3.1 Participant characteristics

Because of data relevance to interpreting changes over the course of the study, previous results (see Tables and Figures) are shown here for ease of the reader (Gilbertson et al., 2020a, 2020b). Seventeen adults met eligibility criteria and were match‐paired to SC (*n* = 7) or EX + SC (*n* = 10), but three subjects were excluded due to non‐compliance to the exercise intervention (*n* = 1 EX + SC), failure to obtain blood post‐intervention due to IV difficulty (*n* = 1 EX + SC), and inability to complete the intervention prior to surgery (*n* = 1 EX + SC). Patient characteristics including age (SC 39.0 ± 5.3 vs. EX + SC 45.6 ± 4.8 years), sex (SC, *n* = 6 females, *n* = 1 male vs. EX + SC, *n* = 7 females), race/ethnicity (SC, *n* = 5 Caucasians, *n* = 2 African Americans vs. EX + SC, *n* = 5 Caucasians, *n* = 1 African American, *n* = 1 Pacific Islander), and type of surgical procedure (SC, *n* = 3 RYGB, *n* = 4 SG vs. EX + SC, *n* = 3 RYGB, *n* = 4 SG) were similar for SC and EX + SC (all, *p *> 0.37).

### 3.2 Body composition and fitness

There were no significant differences in participants performing SC vs. EX + SC for changes in body weight, waist circumference, or body composition from surgery to 30 days post‐surgery. However, SC had a large effect (ES ≥ 1.45) and significantly (all *p *= 0.02) greater improvements in the change in VO_2_peak from time of surgery to post‐surgery than EX + SC (Table 1). VO_2_peak (L/min) was significantly (*p *= 0.05) higher 30 days post‐surgery for SC than EX + SC (Table 1).

**TABLE 1:** Effect of standard care (SC) and aerobic exercise combined with standard care (EX + SC) on cardiometabolic health outcomes

	SC			EX + SC					Effect size Cohen's *d*	
	Baseline	Surgery	30 days post‐surgery	Baseline	Surgery		30 days post‐surgery			
Body composition										
Weight (kg)	128.4 ± 10.9	127.8 ± 10.3	117.5 ± 9.7	116.5 ± 11.9		116.0 ± 11.8		105.7 ± 10.9		0.04
BMI (kg/m^2^)	46.4 ± 3.0	46.2 ± 2.7	42.4 ± 2.6	43.9 ± 4.2		43.7 ± 4.1		39.8 ± 3.9		0.16
Waist circumference (cm)	138.5 ± 8.5	137.5 ± 8.3	130.5 ± 7.4	126.6 ± 7.8		125.8 ± 7.2		123.9 ± 22.2		0.60
FFM (kg)	60.3 ± 3.3	58.8 ± 3.2	56.4 ± 3.3	54.7 ± 5.1		54.1 ± 4.4		51.6 ± 4.4		0.15
% Body fat	51.7 ± 2.4	52.8 ± 2.4	50.9 ± 2.5	52.3 ± 1.4		52.2 ± 1.8		50.5 ± 1.9		0.23
Fitness										
VO_2_peak (L/min)	2.51 ± 0.14	2.36 ± 0.13	2.4 ± 0.1	2.33 ± 0.17		2.31 ± 0.11		2.0 ± 0.1†,*		1.54
VO_2_peak (mL/kg/min)	20.4 ± 1.5	19.2 ± 1.6	20.9 ± 1.7	20.5 ± 1.3		21.1 ± 2.0		19.5 ± 1.6†		1.45
Blood pressure										
HR (bpm)	70.1 ± 4.9	75.3 ± 5.5	71.7 ± 5.1	85.4 ± 5.4		86.9 ± 4.0		78.7 ± 5.9		0.53
Systolic BP (mmHg)	121.4 ± 8.9	122.5 ± 9.1	115.1 ± 6.8	131.5 ± 6.5		134.2 ± 5.6		120.8 ± 6.4		0.61
Diastolic BP (mmHg)	66.0 ± 4.7	63.8 ± 4.5	64.2 ± 4.1	73.4 ± 4.0		75.6 ± 2.7		72.6 ± 4.6		0.34
Aortic waveforms (%)										
Fasted AIx@75	33.3 ± 5.9	32.3 ± 6.1	32.9 ± 7.8	41.3 ± 6.4		27.8 ± 4.2		24.8 ± 4.3		0.79
120 min AIx@75	34.8 ± 8.0	32.6 ± 5.4	15.6 ± 6.5	29.0 ± 5.9		21.8 ± 5.4		29.3 ± 4.8^†,^ *		1.78
AIx@75 120min tAUC	3696 ± 456	3486 ± 801	2874 ± 731	4274 ± 591		3370 ± 587		3165 ± 330		0.31
Adipokines (ng/mL)										
Leptin^||^	100.4 ± 17.2	105.0 ± 20.9	49.3 ± 7.3	90.9 ± 13.5		87.8 ± 18.5		64.7 ± 24.8		0.89
HMW Adiponectin	925 ± 150.6	995 ± 161	1153 ± 149	2375 ± 608		2240 ± 521		2581 ± 405*		0.08
Total Adiponectin^||^	4305 ± 310	4317 ± 275	3639 ± 205	6216 ± 1252		5361 ± 1063		5342 ± 870		0.77
HMW: Total	0.22 ± 0.02	0.23 ± 0.02	0.34 ± 0.04	0.34 ± 0.04		0.38 ± 0.04		0.46 ± 0.03 *		0.21
HMW: Leptin	13.6 ± 3.6	14.6 ± 3.7	35.5 ± 8.8	29.3 ± 8.7		30.1 ± 7.6		65.4 ± 18.7		0.54
Inflammation										
hs‐CRP (μg/ml)	4.95 ± 1.27	5.82 ± 1.26	5.04 ± 1.45	4.54 ± 1.26		4.38 ± 1.15		4.03 ± 1.43		0.18
CK18 (U/L)	189.3 ± 52.1	211.4 ± 64.0	162.3 ± 42.0	191.6 ± 34.5		146.4 ± 24.1		140.5 ± 25.0		0.54
Blood substrates										
Fasting glucose (mg/dL)	100.0 ± 2.4	95.8 ± 4.4	86.0 ± 3.0	101.2 ± 5.4		105.3 ± 6.0		95.0 ± 3.5		0.42
120 min Glucose (mg/dL)	96.9 ± 4.1	95.4 ± 3.8	84.4 ± 4.7	107.9 ± 8.2		105.7 ± 3.8		95.8 ± 4.0		0.39
Glucose tAUC (mg/dL●120min)	13497 ± 739	12687 ± 424	11978 ± 762	15015 ± 1277		14501 ± 1160		14728 ± 1309		0.44
Fasting insulin (μU/mL)	13.4 ± 2.9	11.6 ± 2.0	5.0 ± 1.6	8.5 ± 1.8		10.4 ± 3.0		6.1 ± 1.4		0.52
120 min insulin (μU/mL)	19.0 ± 6.0	13.2 ± 3.1	5.8 ± 1.0	13.4 ± 4.7		12.1 ± 4.4		5.7 ± 1.5		0.13
Insulin tAUC (μU/ml●120 min)	3859 ± 541	3736 ± 961	4448 ± 1042	2932 ± 562		3093 ± 717		2413 ± 331		1.02
HDL cholesterol (mg/dL)	40.9 ± 3.8	42.7 ± 4.6	31.7 ± 2.2	47.8 ± 4.3		45.2 ± 2.6		37.3 ± 2.2		0.37
LDL cholesterol (mg/dL)	139.6 ± 7.1	133.1 ± 15.7	111.1 ± 7.6	134.3 ± 5.7		129.8 ± 7.1		100.3 ± 7.9		0.25
Total cholesterol (mg/dL)	200.7 ± 7.3	195.4 ± 21.7	160.6 ± 7.6	201.2 ± 9.0		192.3 ± 7.2		158.7 ± 8.4		0.03
Triglycerides (mg/dL)	122.9 ± 30.2	118.3 ± 23.0	106.6 ± 6.5	114.4 ± 16.3		110.4 ± 29.7		121.6 ± 18.7		0.29
Substrate metabolism										
0 min RER	0.78 ± 0.02	0.79 ± 0.04	0.75 ± 0.03	0.77 ± 0.02		0.75 ± 0.01		0.71 ± 0.004		0.00
120 min RER	0.82 ± 0.02	0.78 ± 0.02	0.72 ± 0.01	0.78 ± 0.02		0.76 ± 0.02		0.74 ± 0.02		0.63
Metabolic flexibility	0.04 ± 0.01	−0.2 ± 0.02	−0.03 ± 0.02	0.02 ± 0.01		0.03 ± 0.01		0.02 ± 0.02		0.07
Insulin sensitivity^||^	5.22 ± 0.67	6.80 ± 1.82	11.85 ± 2.95	8.49 ± 2.15		10.71 ± 3.41		10.86 ± 2.88		0.77

Abbreviations: Body mass index (BMI); fat free mass (FFM); heart rate (HR); blood pressure (BP); augmentation index (AIx@75); total area under the curve (tAUC); high molecular weight (HMW); high sensitivity C‐reactive protein (hs‐CRP); cytokeratin‐18 (CK18); high‐density lipoproteins (HDL); low‐density lipoproteins (LDL); respiratory exchange ratio (RER); body weight (BW). Data are means ± SEM.

† Significant (*p* < 0.05) difference between treatments in change from surgery to 30 days post‐surgery.

* Significant (*p* < 0.05) 30‐day post‐surgery difference between treatments.

|| Non‐normally distributed data are presented in raw version for ease of interpretation. Fasted AIx@75 (SC *n* = 7, EX + SC *n* = 5), total adiponectin (SC *n* = 6, EX + SC *n* = 7), HMW adiponectin (SC *n* = 6, EX + SC *n* = 7), HMW: total (SC *n* = 7, EX + SC *n* = 6), fasting glucose (SC *n* = 7, EX + SC *n* = 6), 120 min glucose (SC *n* = 7, EX + SC *n* = 6), HDL cholesterol (SC *n* = 7, EX + SC *n* = 6), LDL cholesterol (SC *n* = 7, EX + SC *n* = 6), total cholesterol (SC *n* = 7, EX + SC *n* = 6), 0 minute RER (SC *n* = 7, EX + SC *n* = 6), insulin sensitivity (SC *n* = 5, EX + SC *n* = 7). Conversions: glucose, 1.00 mmol/L = 18.01 mg/dL; insulin, 1.00 μU/mL = 6.95 pmol/L; adipokines, 1.00 ng/mL = 0.001 μg/mL.

### 3.3 Blood pressure and pulse wave analysis

There were no significant differences in those undergoing SC vs. EX + SC for heart rate, blood pressure, or AIx@75 30 days post‐surgery (Table 1). Individuals performing EX + SC had a medium effect (ES ≥ 0.53) for reductions in the change in heart rate, systolic BP, and fasted AIx@75 compared to SC from surgery to 30 days post‐surgery (Table 1). SC participants had a significantly (*p *= 0.02) greater reduction in 120 min AIx@75 from surgery to 30 days post‐surgery compared to EX + SC (Table 1).

### 3.4 Adipokines and inflammation

EX + SC participants had significantly higher HMW adiponectin (*p *= 0.01) and ratio of HMW to total adiponectin (*p *= 0.04) than SC (Table 1) 30 days after surgery. EX + SC adults had a medium effect (ES = 0.54) for the increase in the ratio of HMW adiponectin to leptin compared to SC from surgery to 30 days post‐surgery. SC adults had a medium effect (ES ≥ 0.54) vs. EX + SC for decreased leptin and CK18 from surgery to 30 days post‐surgery (Table 1).

### 3.5 Blood substrates and substrate oxidation

There was no statistical difference for glycemia, circulating insulin or substrate metabolism changes from surgery to 30 days post‐surgery or at 30 days post‐surgery between groups, despite those undergoing SC having medium effect (ES = 0.77) for increased insulin sensitivity compared to EX + SC from time of surgery to 30 days post‐surgery (Table 1).

### 3.6 Quality of life, dietary intake, and physical activity

Individuals performing SC had a significantly (*p *= 0.05) greater increase in the symptoms domain from surgery to 30 days post‐surgery compared to those in EX + SC (Table 2). Participants enrolled in SC had a large effect size (ES = 1.24) and significantly (*p *= 0.04) greater reductions in unsaturated fat intake from surgery to 30 days post‐surgery than EX + SC (Table 3). There were no other differences between groups, although those undergoing SC also had medium effects (ES ≥ 0.52) for decreasing total calorie, carbohydrate, sugar, and water intake from surgery to 30 days post‐surgery (Table 3). During the presurgical intervention, EX + SC participants completed about 19 exercise sessions for nearly 37 min/session at approximately 75% HR_peak_ during the intervention (Gilbertson et al., 2020a, 2020b). From surgery to 30 days post‐surgery, those in SC generally had increased sedentary behavior and reduced time in light physical activity vs. EX + SC, which had a large effect size (ES = 1.86) and significantly (*p *= 0.006) greater decreases in MVPA than SC (Table 3).

**TABLE 2:** Effect of standard care (SC) and aerobic exercise combined with standard care (EX + SC) on weight related quality of life (QoL)

	SC			EX + SC		
	Baseline	Surgery	30 days post‐surgery	Baseline	Surgery	30 days post‐surgery
Symptoms	61.4 ± 7.1	64.5 ± 6.1	73.3 ± 6.2	64.2 ± 7.5	78.0 ± 4.0	74.8 ± 3.1†
Sexual life	58.2 ± 7.4	56.1 ± 7.7	67.4 ± 4.4	57.1 ± 9.4	71.4 ± 4.4	72.6 ± 5.7
Activity/mobility	71.7 ± 8.9	68.0 ± 7.7	79.1 ± 6.1	64.6 ± 8.7	75.1 ± 7.7	69.2 ± ± 4.6
Hygiene/clothing	71.2 ± 6.2	69.8 ± 6.5	79.6 ± 5.6	74.3 ± 3.2	75.5 ± 5.6	83.3 ± 3.1
Emotions	50.6 ± 5.8	53.6 ± 6.5	64.9 ± 3.0	49.9 ± 4.5	63.8 ± 6.3	62.9 ± 6.4
Social interactions	68.2 ± 6.7	67.1 ± 6.2	73.2 ± 4.5	58.9 ± 6.7	70.9 ± 4.4	75.3 ± 5.1
Total score	62.9 ± 5.4	63.0 ± 5.4	72.8 ± 3.4	60.2 ± 5.5	72.1 ± 4.3	71.6 ± 3.8

Data are means ± SEM.

† Significant (*p* < 0.05) difference between treatments in change from surgery to 30 days post‐surgery.

**TABLE 3:** Effect of standard care (SC) and aerobic exercise combined with standard care (EX + SC) on dietary intake and non‐exercise physical activity

	SC			EX + SC			Effect size Cohen's *d*
	Baseline	Surgery	30 days post‐surgery	Baseline	Surgery	30 days post‐surgery	
Dietary intake							
Calories	1922 ± 273	1827 ± 414	581 ± 116	1987 ± 232	1240 ± 286	539 ± 90	0.52
Carbohydrates (g)^||^	182.9 ± 29.7	202.7 ± 54.9	52.3 ± 10.3	228.1 ± 30.6	119.6 ± 33.5	52.8 ± 5.6	0.77
Fiber (g)	13.0 ± 2.2	8.6 ± 0.5	4.7 ± 1.1	20.4 ± 2.7	9.4 ± 1.5	6.0 ± 1.2	0.13
Sugar (g)	66.0 ± 15.1	62.9 ± 6.7	19.4 ± 6.1	69.5 ± 7.8	42.3 ± 12.7	19.6 ± 2.2	0.77
Protein (g)^||^	112.0 ± 24.7	112.4 ± 37.7	44.3 ± 11.6	82.0 ± 10.6	68.5 ± 11.8	34.6 ± 8.4	0.40
Fat (g)	82.9 ± 10.7	62.3 ± 10.5	22.6 ± 6.0	87.7 ± 9.7	49.5 ± 14.6	22.8 ± 5.9	0.31
Saturated Fat (g)	26.0 ± 3.0	18.0 ± 3.5	9.7 ± 2.8	24.8 ± 3.0	16.5 ± 6.3	6.0 ± 1.3	0.13
Unsaturated Fat (g)	25.3 ± 5.9	25.7 ± 6.5	6.5 ± 1.8	29.0 ± 4.6	11.4 ± 2.9	7.2 ± 1.7†	1.24
Trans fat (g)	0.80 ± 0.21	0.42 ± 0.16	0.28 ± 0.10	0.31 ± 0.07	0.18 ± 0.10	0.07 ± 0.03	0.09
Cholesterol (mg)	426.0 ± 98.1	295.6 ± 87.0	170 ± 65	307.0 ± 70.2	171.3 ± 52.2	106 ± 31	0.22
Water (g)	2195 ± 463	2323 ± 637	1230 ± 164	1539 ± 296	1570 ± 390	1128 ± 286	0.59
Physical activity (%)							
Sedentary NEPA time	26.1 ± 4.0	26.0 ± 5.6	49.0 ± 7.6	38.5 ± 2.3	41.5 ± 5.8	50.3 ± 3.7	0.80
Sedentary total time	26.1 ± 4.0	26.0 ± 5.6	49.0 ± 7.6	38.5 ± 2.3	39.0 ± 5.2	50.3 ± 3.7	0.67
Light NEPA time	69.5 ± 3.5	70.1 ± 6.3	48.8 ± 7.6	54.6 ± 2.4	52.7 ± 5.3	45.1 ± 3.8	0.78
Light total time	69.5 ± 3.5	70.1 ± 6.3	48.8 ± 7.6	54.6 ± 2.4	49.8 ± 5.2	45.1 ± 3.8	0.96
MVPA NEPA time	4.4 ± 0.8	3.9 ± 0.9	2.2 ± 0.3	6.9 ± 0.8	5.7 ± 0.9	4.6 ± 0.7	0.27
MVPA total time	4.4 ± 0.8	3.9 ± 0.9	2.2 ± 0.3	6.9 ± 0.8	11.2 ± 1.1	4.6 ± 0.7†	1.86

Abbreviations: Grams (g); milligrams (mg), non‐exercise physical activity (NEPA); moderate to vigorous physical activity (MVPA). Data are means ± SEM.

† Significant (*p* < 0.05) difference between treatments in change from surgery to 30 days post‐surgery.

* Significant (*p* < 0.05) 30‐day post‐surgery difference between treatments.

|| Non‐normally distributed data are presented in raw version for ease of interpretation. Fiber (SC *n* = 5, EX + SC *n* = 7), sugar (SC *n* = 6, EX + SC *n* = 6), trans fat (SC *n* = 7, EX + SC *n* = 6), and physical activity variables (SC *n* = 6, EX + SC *n* = 6).

### 3.7 Correlations

**FIGURE 2:**
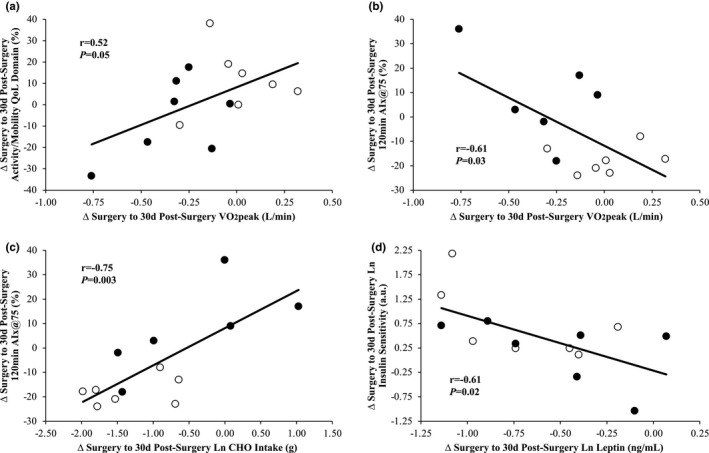
The increase in VO_2_peak was associated with an increase in activity/mobility quality of life (QoL) domain (a) and decrease in 120 min augmentation index (AIx@75) (b) from surgery to 30 days post‐surgery. The decrease carbohydrate (CHO) intake correlated to the decrease in 120 min AIx@75 (c) from surgery to 30 days post‐surgery. The decrease in leptin was associated with an increase in insulin sensitivity (d) from surgery to 30 days post‐surgery. Insulin sensitivity, leptin, and CHO were not normally distributed and therefore variables were log transformed and presented herein. Change, ∆; Open circles, standard care (SC); closed circles, aerobic exercise combined with standard care (EX + SC)

Fat mass reductions correlated with increased HMW adiponectin (*r* = −0.63, *p *= 0.02), the ratio of HMW to total adiponectin (*r* = −0.55, *p *= 0.04), and personal hygiene/clothing QoL domain (*r* = −0.67, *p *= 0.009) from time of surgery to 30 days post‐surgery. Gains in VO_2_peak (L/min) were associated with increased activity/mobility QoL domain (*r* = 0.52, *p* = 0.05; Figure 2A) as well as decreased 120 min AIx@75 (*r* = −0.61, *p *= 0.03; Figure 2B) from time of surgery to 30 days post‐surgery. The decrease in 120 min AIx@75 also correlated to the decrease in carbohydrate intake (*r* = 0.75, *p *= 0.003; Figure 2C) from time of surgery to 30 days post‐surgery. The decrease in 120 min insulin was associated with a decrease in AIx@75 tAUC (*r* = 0.62, *p *= 0.02) from time of surgery to 30 days post‐surgery. The increase in insulin sensitivity was linked to the decrease in leptin (*r* = −0.61, *p *= 0.02; Figure 2D) from surgery to 30 days post‐surgery. The increase in total time spent in MVPA was associated with a reduction in CK18 (*r* = −0.67, *p *= 0.02) from time of surgery to 30 days post‐surgery.

## 4 DISCUSSION

Participants undergoing EX + SC improved heart rate, systolic blood pressure, fasted AIx@75, and the ratio of HMW adiponectin to leptin, based on effect sizes, from time of surgery to 30 days post‐surgery to a greater extent than SC. However, contrary to our hypothesis, participants undergoing EX + SC did not have significantly greater improvements in any other cardiometabolic health or QoL domain after surgery compared to SC. These findings are somewhat surprising because prior prehabilitation studies have shown that aerobic exercise enhances postoperative outcomes including functional capacity, pain, and QoL 1 month after elective surgeries compared to standard medical practice (Richardson et al., 2017; Valkenet et al., 2011). One possible reason for this lack of maintenance/improvement in cardiometabolic health post‐surgery may relate to declines in fitness seen within the EX + SC treatment group. In fact, the present study found that patients in SC had significant improvements in relative measures of VO_2_peak compared to reductions in fitness measures in patients assigned to EX + SC 30 days post‐surgery. These findings are noteworthy since participants undergoing EX + SC had a significantly greater reduction in total time spent in MVPA compared to SC after surgery. Interestingly, the gain in VO_2_peak occurred despite those in SC having greater increases in sedentary behavior and declines in light physical activity compared to individuals in EX + SC from the time of surgery to 30 days post‐surgery. In either case, decreasing time spent in MVPA has been directly linked to diminishes in VO_2_peak and decreased QoL (Bouchard et al., 2015). In support of this, we report that decreased VO_2_peak was associated with a reduction in the QoL domain activity/mobility (Figure 2A). Collectively, these findings suggest that individuals in EX + SC did not maintain aerobic exercise after bariatric surgery. This reduction in MVPA from before surgery may have promoted both declines in aerobic fitness as well as the inability to complete activities of daily living that, in turn, may have led to medical symptoms with no apparent cause (Donini et al., 2020). Future work should determine if maintaining and/or increasing postoperative VO_2_peak can preserve QoL seen during surgical prehabilitation (Gilbertson et al., 2020a, 2020b).

Participants in EX + SC had significantly higher HMW adiponectin and ratio of HMW to total adiponectin than SC 30 days after surgery. Our prior work found that those in EX + SC significantly increased the ratio of HMW to total adiponectin compared to no change with SC from pre‐intervention to post‐intervention (i.e., time of surgery), and the increase in this ratio was associated with increased VO_2_peak and decreased fat mass (Gilbertson et al., 2020b). Findings in the present study are similar in that the decrease in fat mass correlated to an increase in HMW adiponectin and the ratio of HMW to total adiponectin from the time of surgery to 30 days post‐surgery. Together, these findings herein suggest that the preoperative exercise can maintain effects on adiponectin up to 30 days after bariatric surgery despite changes in MVPA. The clinical relevance of these findings are unclear though despite adiponectin having beneficial cardiovascular effects (van Andel et al., 2018; Pischon et al., 2011). Exercise did not lead to overall improvements in cardiometabolic health post‐surgery. In fact, decreases in total time spent in MVPA from the time of surgery to 30 days post‐surgery was associated with increases in CK18. While our prior work demonstrated that EX + SC elicited significant reductions in CK18 prior to surgery compared to SC (Gilbertson et al., 2020a), the current wok suggests removing exercise led to elevations in CK18. Concomitantly, we show that the decrease in leptin from surgery to 30 days post‐surgery was also related to increased insulin sensitivity. This supports that leptin is an important hormone for regulating glucose metabolism (Ceddia, 2005), and these findings raise the hypothesis that pre‐ and postoperative exercise are likely needed to optimize the health and well‐being of patients undergoing bariatric surgery.

In the present work we report that patients undergoing SC had a greater reduction in postprandial AIx@75 than EX + SC from surgery to 30 days post‐surgery and at 30 days post‐surgery. These findings contrast our prior work suggesting that EX + SC had a modest effect size for decreasing 120 min AIx@75 and AIx@75 tAUC compared to SC during the preoperative period in patients receiving bariatric surgery (Gilbertson et al., 2020a). However, in this previous work EX + SC increased VO_2_peak pre‐surgery. Supporting the role of fitness on aortic waveforms, we show herein that the rise in VO_2_peak was associated with a decrease in 120 min AIx@75 (Figure 2B). Thus, a fitness‐related mechanism including, but not limited to, decreased sympathetic input, reduced endothelium‐derived vasoconstrictor molecules, and/or increased nitric oxide availability may relate to improvements in postprandial aortic waveforms (Gando et al., 2016; Tanaka, 2019; Vaitkevicius et al., 1993), although these were not evaluated in the present study and should be considered in future work. Interestingly, we also show that the decrease in 120 min AIx@75 correlated with decreased carbohydrate intake (Figure 2C) from surgery to 30 days post‐surgery. This is consistent with recent work by our group in which caloric restriction, with or without exercise, improves postprandial AIx@75 in women with obesity (Heiston et al., 2020). A decrease in carbohydrate intake elicits a smaller release of insulin in the postprandial state. Because SC had a greater effect for increased insulin sensitivity than EX + SC, it is possible that improved insulin action may have contributed to arterial health (Jia et al., 2015).

There are limitations to the study that require acknowledgement. Due to the small sample size (*n* = 14) these findings should be considered pilot data. Additionally, this study may be underpowered to detect statistical differences in some outcomes. For example, it appears that patients performing exercise before surgery had modest effect size improvements in several cardiovascular measures, including heart rate, blood pressure, and fasting AIx@75 when compared to those patients undergoing SC. Subsequently, larger studies are needed to confirm the results presented in the current work. Nearly all of our participants were females (*n* = 13), and therefore our findings may not be generalizable to males receiving bariatric surgery. There is potential for variation in cardiometabolic health outcomes 30 days after surgery based on the type of bariatric surgery (RYGB or SG), as RYGB elicits greater improvements in body weight and insulin sensitivity than SG 30 days after bariatric surgery (Karamanakos et al., 2008; Lager et al., 2017; Samat et al., 2013). Nevertheless, participants were match‐paired based off surgery type to minimize this concern. Another limitation is that aerobic exercise sessions were not supervised in the present study. Research shows that there is large variability in adherence to unsupervised exercise sessions in obese populations (Colley et al., 2008). Thus, supervised aerobic exercise may have elicited greater changes in physiological and psychological outcomes than unsupervised aerobic exercise (Lacroix et al., 2017; Vemulapalli et al., 2015). However, participants in the present study were required to complete 80% of exercise sessions, and the research team conducted weekly check‐ins during the intervention to ensure adherence to the exercise intervention.

## 5 CONCLUSIONS

In summary, this exploratory project was undertaken to determine if preoperative exercise‐ mediated adaptation would induce differential health changes in post‐surgery cardiometabolic health as well as if increased physical activity/exercise behavior before surgery would persist post‐surgery. The present study showed that preoperative EX + SC did not elicit greater overall improvements in cardiometabolic health and QoL than SC alone in the 30‐day postoperative period, despite some sustained benefit in blood pressure, resting heart rate, HMW adiponectin to total adiponectin as well as leptin ratio, and fasted AIx@75. Surprisingly, individuals undergoing SC had greater improvements in aerobic fitness, postprandial aortic waveforms, as well as the inflammatory markers (CK18 and leptin) than EX + SC. This is likely related to declines in MVPA observed in the EX + SC group due to these participants not maintaining their preoperative exercise in the post‐surgical period. These findings therefore provide unique insight to the potential of physical activity/exercise to modify health and well‐being in patients undergoing bariatric surgery. Additional work is warranted to understand optimal exercise recommendations before, immediately after and long‐term in bariatric patients to reduce chronic disease recidivism.

## CONFLICT OF INTEREST

The authors declare no potential or perceived financial or other conflict of interest.

## AUTHOR CONTRIBUTIONS

SKM conceptualized and designed the study. NMG, NZME, JMG, MK, EAR, PTH, and SKM recruited participants and collected data. NMG and SKM analyzed data. All authors interpreted data. NMG and SKM were primarily responsible for writing the manuscript, and all authors edited the manuscript. All authors approved submission.
